# The Nomenclature of Diseases

**Published:** 1918-09-14

**Authors:** 


### The Hospital

The Workers' Newspaper of Administrative Medicine and Institutional Life, Administration, National Insurance and Health.

No. 1684, Vol. LXIV. SATURDAY, SEPTEMBER 14, 1918. PRICE TWOPENCE

### THE NOMENCLATURE OF DISEASES.

In affairs generally, and particularly perhaps when questions of statistics are concerned, an identical use of names is a matter of high importance. Clearly, unless the persons concerned in the issue use each of the terms employed in an agreed and determined sense, misunderstandings are more than possible, and the discussion, though professedly about one and the same thing, may really be about quite different things. It is one of the first conditions of useful debate that there shall be preliminary agreement on the definition of the terms to be used in the argument. Similarly, when various observers undertake to record their individual experience there is need of an accepted scheme of classification in which each of the various groups and classes shall have, to those who employ them, a common and accepted value. Otherwise the statistical results will be unreliable and valueless and misleading. What is true in this respect as a general claim has a special application to medical science. Here there is much that is fluid and uncertain, with a corresponding opportunity for vagueness and conflict in the use of names, and for the development of personal peculiarities in the habits of individual observers. Thus it is notorious that the accurate collection of medical statistics is a matter of great difficulty, and that over the figures there hangs a more or less extensive shadow of doubt. To possible inaccuracies of observation there is added the fear that all the experiences which bear one and the same label do not as a matter of fact refer to facts of an identical order. Now, in at least one department of the national polity medical statistics have a high practical value. We refer to the record of the existence and distribution of various forms of disease and to the registration of the causes of death. It is upon this statistical basis that rests a knowledge of the public health, and such knowledge is a necessary first step in any scheme for the prevention of disease. Therefore, whatever will promote uniformity on the part of medical practitioners in the use of names applied to various diseased conditions is both good in itself and of value to the common interest.

For such general reasons as those above stated the issue by the Royal College of Physicians of a Nomenclature of Diseases is worthy of attention. In form the volume has anything but an inviting character, and even the most rigid enthusiast for accuracy and precision will not find relaxation in its pages. It presents itself as a long list of names indicating the diseases which affect either the body as a whole or particular parts of the body, and the amateur in gazing at these might well wonder how any of us contrives to keep alive. But the provision of light reading is not its object. Its aim is to secure uniformity of record, and this particularly in the registration of the causes of death. The list has no sanction in the shape of compulsion, but it has some educative force. Moreover, through it pressure may be brought to bear on those who tend to laxity in statement or to an individual nonconformity. Where it touches the public interest is the moral demand that every certificate of the cause of death shall be phrased in harmony with its terms, and thus there is some chance of arranging in appropriate order the statistics which it is the duty of the Registrar-General to provide. Assuming the observations to be accurate, here is the appropriate fashion in which they may be recorded with a view to scientific and practical presentation.

At first sight it might seem a comparatively simple thing to arrange in order the names of the various diseases to which the different parts of the human body are liable. But there are more difficulties than those which appear on the surface. Completeness of enumeration and strict accuracy are essential, and to obtain these in every part of the field means much painstaking inquiry and investigation. The question of synonyms also is of great importance, and here again much knowledge and a nice judgment are required. The present issue of the Nomenclature was begun in 1912 and, making all allowance for the difficulties just stated and for the exceptional demands of the last four years, it cannot be said that the work has been marked by expedition. There is, however, more than a full precedent, for the first edition of the volume, though begun in 1859, did not make its appearance until ten years later. It is due to the College, and particularly to the special committee charged with the several departments of the work, to recognise that in the issue of this volume service is rendered to the public interest, and that the service is a laborious one and little likely to attract either recognition or reward.

When the scheme of classification adopted by the College is considered it is at once seen how very far from scientific precision is our present knowledge of practical medicine. Probably the best classification of diseases would be an etiological one. But such an enterprise at present is entirely out of the question. The diseases of which the pathogenesis is accurately known are relatively few, and though knowledge in this direction is extending it has still much ground to compass. We have to be content for practical purposes with something short of logical completeness. On many sides medical knowledge is in a state of transition. The old confident clinical diagnoses do not satisfy the modern passion for a knowledge of causes and until this knowledge is reached some degree of mental unrest is inevitable. So far from its being an experience to be deplored it is to be accepted as a note of life and interest. But if, in the transition period, certainty of knowledge is not possible, accuracy and uniformity in the use of names and phrases can be cultivated. The carefully and critically prepared Nomenclature of Diseases issued by the College of Physicians is an aid to these desirable ends, and it merits, therefore, gratitude and good will.

